# Unique metabolism and protein expression signature in human decidual NK cells

**DOI:** 10.3389/fimmu.2023.1136652

**Published:** 2023-03-03

**Authors:** Ping Wang, Tingting Liang, Heqin Zhan, Mingming Zhu, Mingming Wu, Lili Qian, Ying Zhou, Fang Ni

**Affiliations:** ^1^ Department of Hematology, The First Affiliated Hospital of University of Science and Technology of China (USTC), The Chinese Academy of Science (CAS) Key Laboratory of Innate Immunity and Chronic Disease, School of Basic Medical Sciences, Division of Life Sciences and Medicine, University of Science and Technology of China, Hefei, China; ^2^ Institute of Immunology, University of Science and Technology of China, Hefei, China; ^3^ Department of Pathology, School of Basic Medical Sciences, Anhui Medical University, Hefei, China; ^4^ Department of Obstetrics and Gynecology, The First Affiliated Hospital of University of Science and Technology of China (USTC), Division of Life Sciences and Medicine, University of Science and Technology of China, Hefei, China

**Keywords:** metabolomics, proteomics, natural killer cell, decidua, glycerophospholipid, glutathione

## Abstract

Human decidual natural killer (dNK) cells are a unique type of tissue-resident NK cells at the maternal-fetal interface. dNK cells are likely to have pivotal roles during pregnancy, including in maternal-fetal immune tolerance, trophoblast invasion, and fetal development. However, detailed insights into these cells are still lacking. In this study, we performed metabolomic and proteomic analyses on human NK cells derived from decidua and peripheral blood. We found that 77 metabolites were significantly changed in dNK cells. Notably, compared to peripheral blood NK (pNK) cells, 29 metabolites involved in glycerophospholipid and glutathione metabolism were significantly decreased in dNK cells. Moreover, we found that 394 proteins were differentially expressed in dNK cells. Pathway analyses and network enrichment analyses identified 110 differentially expressed proteins involved in focal adhesion, cytoskeleton remodeling, oxidoreductase activity, and fatty acid metabolism in dNK cells. The integrated proteomic and metabolomic analyses revealed significant downregulation in glutathione metabolism in dNK cells compared to pNK cells. Our data indicate that human dNK cells have unique metabolism and protein-expression features, likely regulating their function in pregnancy and immunity.

## Introduction

Natural killer (NK) cells are innate lymphocytes that can instantly eliminate stressed, infected, transformed, or allogeneic cells without being exposed to them first (1). As the first identified subset of innate lymphoid cells (ILCs), the cytotoxic capacity of NK cells and their capacity to generate cytokines in response to stimulation are their most distinguishing features (2, 3). In addition, NK cells influence other immune cells and are vital in resistance to intracellular bacterial infections, malignancies, and viruses (2, 4, 5). Human NK cell phenotypic characteristics are determined by the expression of CD56 and the absence of CD3, and they can be further subdivided into two major subsets based on the density of CD56 and CD16 expression: CD56^dim^CD16^+^ and CD56^bright^CD16^-^ (6). CD56^dim^CD16^+^ cells are more cytotoxic, whereas CD56^bright^CD16^-^ cells are more specialized for immunoregulation and have more immature functions (4, 7, 8). These subsets differ in phenotype, function, and tissue localization (6, 9, 10).

Recent studies have provided insights into tissue-resident NK cells (trNK), which are found in many tissues including the liver, lung, lymph nodes, kidney, and uterus (3, 11, 12). Uterine decidual NK (dNK) cells are critical during pregnancy, functioning in formation of the fetal-maternal interface, remodeling of the maternal spiral arteries, and promoting fetal development (13–16). In early human pregnancy, the hallmark of the decidua is the abundance of NK cells, which constitute ~70% of decidual lymphocytes (13). These dNK cells express high levels of CD56, CD49a, and both inhibitory receptors and immature surface signature molecules (13). In contrast, human peripheral blood NK (pNK) cells express high levels of CD16, and are frequently thought to be more inherently cytotoxic (6), with significant activity in anti-virus and anti-tumor immunity (5, 17, 18). Despite emerging characterization of phenotypic and functional differences between dNK and pNK cells (14, 19–21), the mechanism behind these differences in human NK cell function is still largely unknown.

Over the past decade, numerous advances in “-omics”-scale technology, such as microarray technology, RNA-sequencing (RNA-seq), and assay for transposase-accessible chromatin using sequencing (ATAC-Seq), have enriched our understanding of the biology of NK cells (22–25). Additionally, single-cell analysis has revealed three primary subgroups of dNK cells, showing cell type-specific activities and intercellular communication at the maternal-fetal interface (26–28). Our group has also used microRNA microarray technology and single cell RNA-seq to uncover the molecular basis of the different phenotypes and functions of human NK cell subsets (29, 30). Although some additional genes or factors have been identified that contribute to the key aspects of human NK cells, our understanding of the molecular basis of the phenotypes and functions of human NK cells is incomplete.

In this work, we applied metabolomics and proteomics to analyze the metabolome and proteome of human dNK and pNK cells. Metabolomics analysis showed significant changes in metabolic pathways and that glycerophospholipid metabolism was downregulated in dNK cells. Integrative proteomics and metabolomics analysis revealed the disequilibrium of redox in dNK cells. Correlation analyses showed that metabolites have strong correlations with NK functions. Our data revealed global internal metabolic alterations between pNK and dNK cells. Our study provides a new perspective on NK cell phenotype, metabolism, and functional network and is a valuable resource enriching our understanding of metabolic alterations in NK cell subsets.

## Materials and methods

### Samples

All decidual samples from healthy donors undergoing elective abortion in the first trimester between 6 and 12 weeks of gestation were obtained at Anhui Provincial Hospital, Hefei. Peripheral blood samples were collected from age-matched non-pregnant healthy women. This study was approved by the Medical Ethics Committee (No. 2022KY063) of the First Affiliated Hospital of the University of Science and Technology of China. All donors supplied informed consent.

### Isolation of human NK cells from decidua and peripheral blood

The cells were processed within 4 h of collection. Peripheral blood samples were diluted 1:2 in PBS. Mononuclear cells were isolated by Ficoll-Hypaque centrifugation using standard procedures (29). CD3^−^CD56^+^ NK cells were isolated from peripheral blood mononuclear cells with the MACS isolation system according to the manufacturer’s instructions (Miltenyi Biotec). Decidual NK cells were isolated from decidual samples as previously described (29, 31). The cell purity was determined to be >95% by post-purification FACS analysis.

### Proteomics analysis and data processing

Frozen samples were transferred into low protein binding tubes (1.5 ml Eppendorf) and lysed with 300 µL lysis buffer supplemented with 1 mM PMSF with sonication. After sonication, the samples were centrifuged at 15,000 g for 15 min to remove insoluble particles. Protein concentration was determined by BCA and aliquoted for storage at -80°C. The filter-aided sample preparation (FASP) approach was used to decrease and trypsinize the isolated proteins (32). The digested peptides were desalted by the C18-Reverse-Phase SPE Column.

All analyses were performed using a Q-Exactive mass spectrometer (Thermo, USA) equipped with a Nanospray Flex source (Thermo, USA). Samples were loaded and separated in a C18 column (15 cm × 75 µm) on an EASY-nLCTM 1200 system (Thermo, USA). The flow rate was 300 nL/min and the linear gradient was 90 min (0-55 min, 8% B; 55-79 min, 30% B; 79-80 min, 50% B; 80-90 min, 100% B; mobile phase A = 0.1% FA in water and B = 80% ACN/0.1% FA in water). Full MS scans were acquired in the mass range of 300 -1,600 m/z with a mass resolution of 70,000 and the AGC target value was set at 1e6. The ten most intense peaks in MS were fragmented with higher-energy collisional dissociation (HCD) and analyzed by MS/MS. MS/MS spectra were obtained with a resolution of 17,500 with an AGC target of 2e5 and a max injection time of 80 ms. Q-E dynamic exclusion was set for 15.0 s and run under positive mode.

Peak lists were generated from raw data files and searched against the Uniprot Human Protein Database using MaxQuant (Version 1.3.0.5). All peptides and proteins were filtered with false discovery rate (FDR) below 0.01. Label-free protein quantification was carried out using LFQ intensities by MaxQuant 1.5.2.8, and overlapped proteins between replicates were used for the following analysis.

### Metabolomics analysis and data processing

A 250 μL mixture of methanol and water (7/3, vol/vol) was added to each sample. QC samples were prepared by mixing aliquots of all samples for a pooled sample. An Acquity UHPLC system (Waters Corporation, Milford, USA) coupled with an AB SCIEX Triple TOF 5600 System (AB SCIEX, Framingham, MA) was used to analyze the metabolic profiling in both ESI positive and ESI negative ion modes. An Acquity UPLC BEH C18 column (1.7 μm, 2.1 × 100 mm) was employed in both positive and negative modes. The binary gradient elution system consisted of (A) water (containing 0.1% formic acid, v/v) and (B) acetonitrile (containing 0.1% formic acid, v/v), and separation was achieved using the following gradient: 0 min, 5% B; 2 min, 20% B; 4 min, 60% B; 11 min, 100% B; 13 min, 100% B; 13.5 min, 5% B and 14.5 min, 5% B. The flow rate was 0.4 mL/min and the column temperature was 45°C. All samples were kept at 4°C during analysis. The injection volume was 5 μL. Data acquisition was performed in full scan mode (m/z ranges from 70 to 1,000) combined with IDA mode. Parameters of mass spectrometry were as follows: ion source temperature, 550°C (+) and 550°C (−); ion spray voltage, 5,500 V (+) and 4,500 V (−); curtain gas of 35 PSI; declustering potential, 100 V (+) and −100 V (−); collision energy, 10 eV (+) and −10 eV (−); and interface heater temperature, 550°C (+) and 600°C (−). For IDA analysis, the range of m/z was set as 50–1,000, and the collision energy was 30 eV. Peak lists were generated from raw data files and searched against the Uniprot Human Protein Database using MaxQuant (Version 1.5.2.8).

### Data analysis

The Gene Ontology (GO) processes and kyoto encyclopedia of genes and genomes (KEGG) pathways of proteomics data were enriched using the Metascape web-based platform (33). KEGG pathways of metabolomics data were enriched by MetaboAnalyst 5.0 (34). The principal component (PC) contribution plots were generated using the factoextra (v.1.0.7) package and only the top 25 contributions were displayed in the contribution plot. R statistical software (v.4.1.2) was used for the correlation analysis. The P value of the correlation coefficient was computed by the corPvalueStudent function with the WGCNA (v.1.71) package. Networks were created by Cytoscape (v.3.9.1) software (35).

### Flow cytometry

Cells were stained with the following human mAbs purchasing from Biolegend for FACS: anti-CD45 conjugated with fluorescein isothiocyanate (FITC), anti-CD3 conjugated with allophycocyanin-Cy7 (APC-Cy7), and anti-CD56 conjugated with phycoerythrin (PE). CellROX Deep Red Reagent was purchased from Invitrogen as reactive oxygen species (ROS) dye. Hoechst stain was purchased from Beyotime. FACS staining was performed according to the manufacturer’s instructions. The data were analyzed using FlowJo software (Version 10).

## Results

### Differential metabolites between dNK and pNK cells

**Figure 1:**
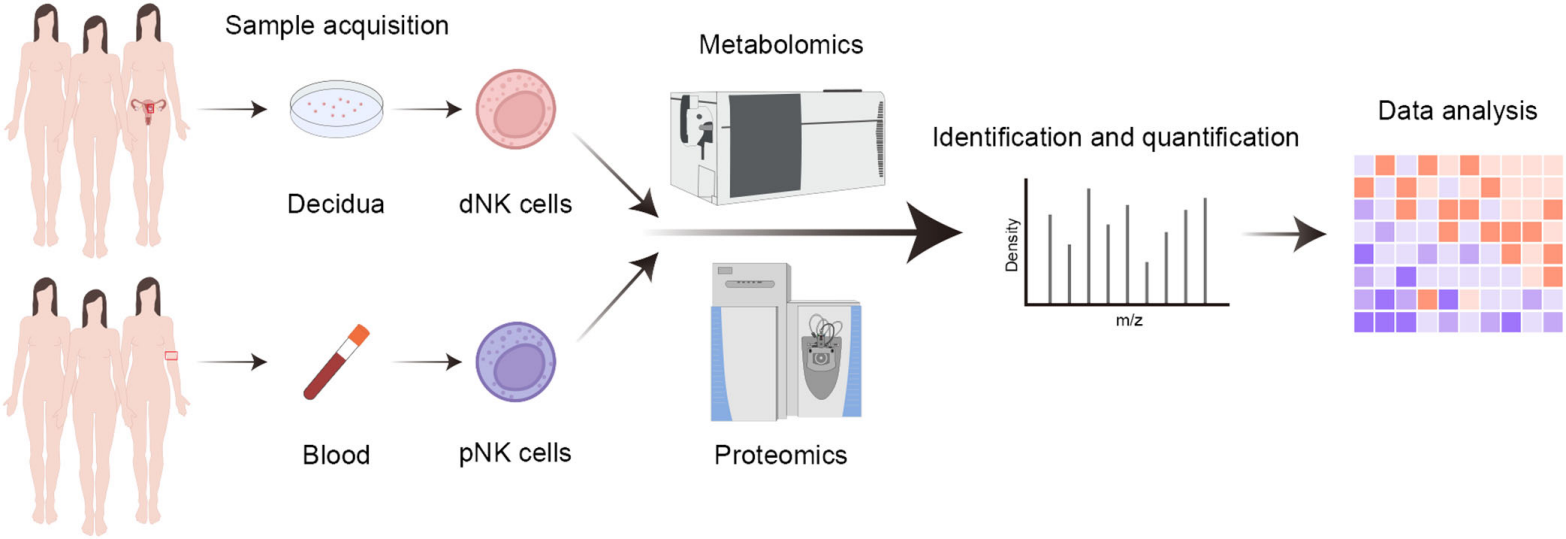
Workflow of proteomics and metabolomics. Summary for the analysis of blood and decidual samples by mass cytometry.

**Figure 2:**
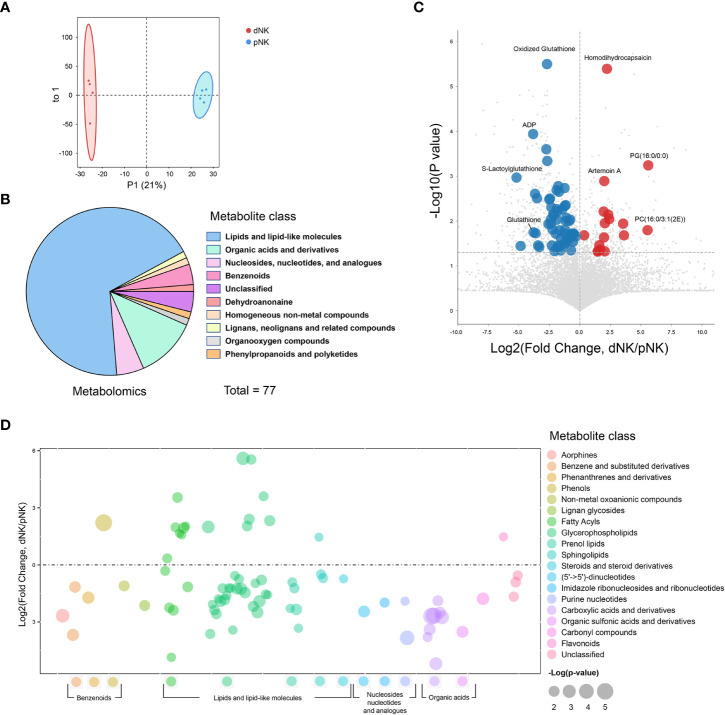
Metabolomics profile of dNK and pNK cells. **(A)** OPLS-DA score plots of metabolomics data. Each symbol represents one donor (n=4). **(B)** Pie chart showing differential metabolites of different classes. Different colors indicate different classes. **(C)** Volcano diagrams showing all of the identified metabolites from metabolomics data. The x-axis and y-axis are based on the fold change (FC) and p-values, respectively. Each dot represents a metabolite. Significantly upregulated, downregulated (VIP > 1, p value < 0.05, FC > 1 or < 1), and unchanged DEPs are colored in red, blue, and gray, respectively. The horizontal line denotes a p-value cutoff of 0.05. **(D)** Bubble plot of log2(fold change) in abundance of metabolite species in dNK relative to pNK cells (ctrl). Values are shown as log2(fold change) relative to pNK cells. Each dot represents a metabolite species. Color-coded per metabolite class. Dot size indicates significance. The horizontal line denotes FC of 1.

We conducted metabolomics analysis on pNK cells from blood (n=4) and dNK cells from humans in the first trimester of pregnancy (n=4) to assess metabolic differences between dNK and pNK cells (**Figure 1**). The orthogonal partial least squares discrimination analysis (OPLS-DA) clearly distinguished dNK from pNK cells (**Figure 2A**). Metabolomics detected 77 differential metabolites (variable importance in projection (VIP) > 1, p value < 0.05) and showed that differential metabolites were mainly composed of lipids, organic acids, nucleosides, and benzenoids (**Figure 2B**). We found 17 metabolites that were upregulated (fold change > 1) and 60 metabolites that were downregulated (fold change < 1) (**Figure 2C**).

To illustrate alterations in different classes of metabolites, we displayed all metabolites in a bubble plot (**Figure 2D**). The bubble plot revealed that the majority of elevated metabolites were found in lipids and lipid-like compounds, particularly fatty acyls and glycerophospholipids, pointing to complex alterations in lipids between pNK and dNK cells (**Figure 2D**). Together, these findings shed light on the differences in metabolite levels between pNK and dNK cells.

### Glycerophospholipid metabolism is downregulated in dNK compared to pNK cells

**Figure 3:**
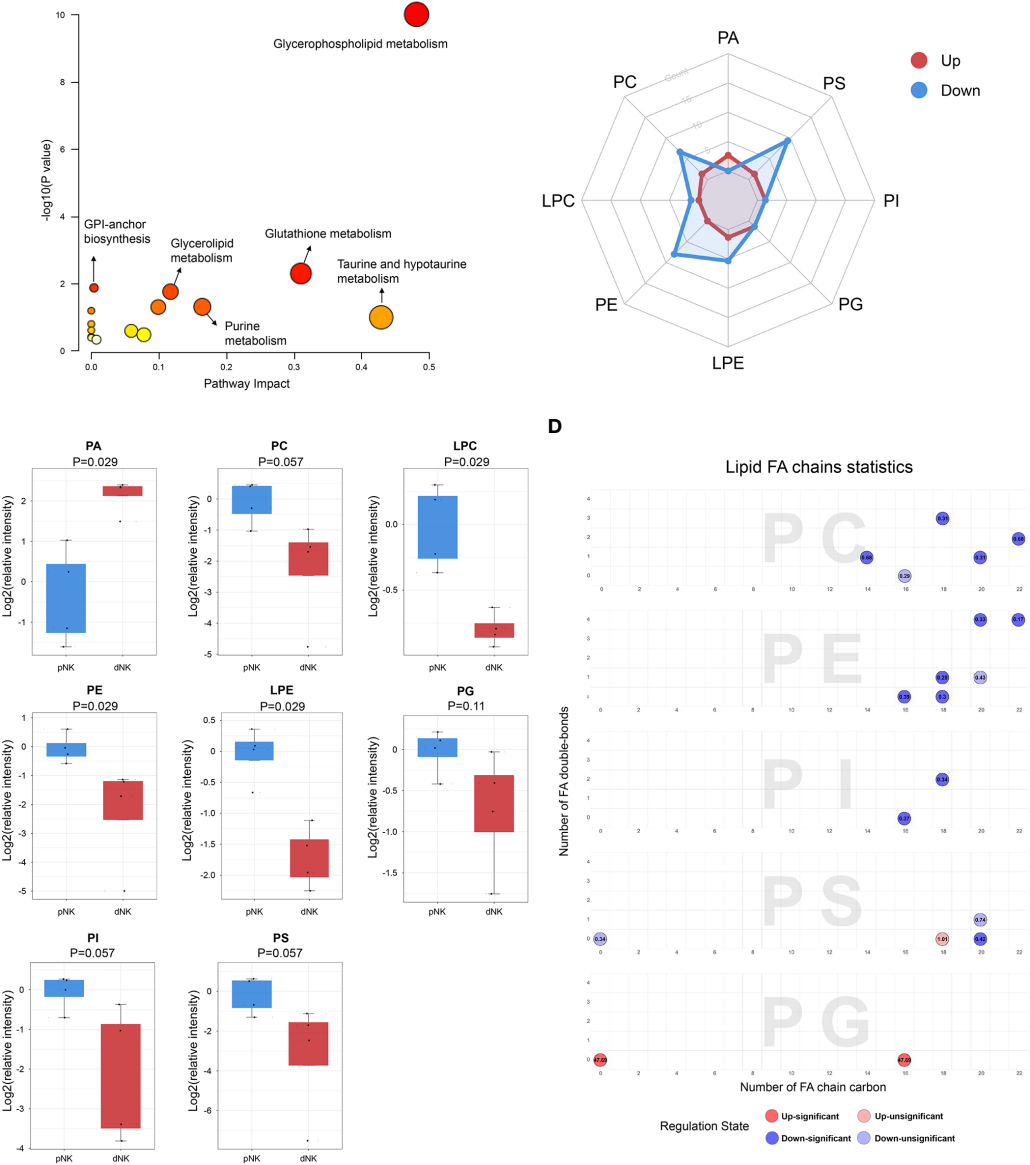
Metabolic pathways change in dNK cells compared to pNK cells. **(A)** KEGG metabolic pathway impact analysis using Metaboanalyst 5.0. **(B)** Radar diagram of subclasses in glycerophospholipids. **(C)** Relative intensity of glycerophospholipid related metabolites in dNK and pNK cells. Statistical analyses were performed using the Mann–Whitney U-test. The box plots show the median and 25^th^ and 75^th^ percentiles, with whiskers indicating maximal and minimal values. **(D)** Lipid fatty acid (FA) chain statistics of glycerophospholipid subclasses. The x-axis presents the number of FA double-bonds and the y-axis presents the number of FA chain carbon. Fold changes are labeled in the circle. The FA chain analysis was conducted using http://www.lintwebomics.info/.

To characterize the differential metabolic pathways between dNK and pNK cells, we performed pathway enrichment analysis of the differential metabolites and observed that glycerophospholipid metabolism, glutathione metabolism, purine metabolism, glycerolipid metabolism, and glycosylphosphatidylinositol (GPI)-anchor biosynthesis were all significantly changed in dNK cells (**Figure 3A**). Glycerolphospholipids, which included 24 differential metabolites, were the most abundantly altered class of metabolites so we analyzed glycerophospholipid subgroups to further determine the alterations. The radar map shows counts of upregulated/downregulated metabolites in these subgroups (**Figure 3B**). Most subgroups had more downregulated metabolites in dNK cells compared to pNK cells except phosphatidic acid (PA). For metabolites contained in each subgroup, phosphatidylcholine (PC), lysophosphatidylcholine (LPC), phosphatidyl-ethanolamine (PE), and lysophosphatidylethanolamine (LPE) were significantly reduced in dNK cells and phosphatidylinositol (PI), phosphatidylserine (PS), and phosphatidylglycerol (PG) showed a trend of reduction (**Figure 3C**), indicating downregulation of glycerophospholipid metabolism in dNK from pNK cells.

A previous study showed that PS exposure was proportional to the degree of NK cell activation during the NK cell activation process (36), suggesting that the reduced cytotoxicity may be linked to the decreased content of glycerophospholipids in dNK cells. In addition to the increase of PA, individual lipids in PG, PC, and PI, such as PG (16:0/0:0), PC (16:0/3:1(2E)), and PI (16:0/16:0) were significantly upregulated in dNK cells (**Supplementary Figure 1**). Plotting chain carbon and double-bonds showed that upregulated glycerophospholipids tended to have fewer double bonds and downregulated glycerophospholipids might have more fatty acid chain carbons in dNK cells (**Figure 3D**). This finding demonstrated that the level of unsaturation of glycerophospholipids was dramatically reduced, implying that dNK cell membrane fluidity may be affected (37). In summary, these results indicate considerable alterations of complex lipids between dNK and pNK cells.

### The metabolic network changed between dNK and pNK cells

**Figure 4:**
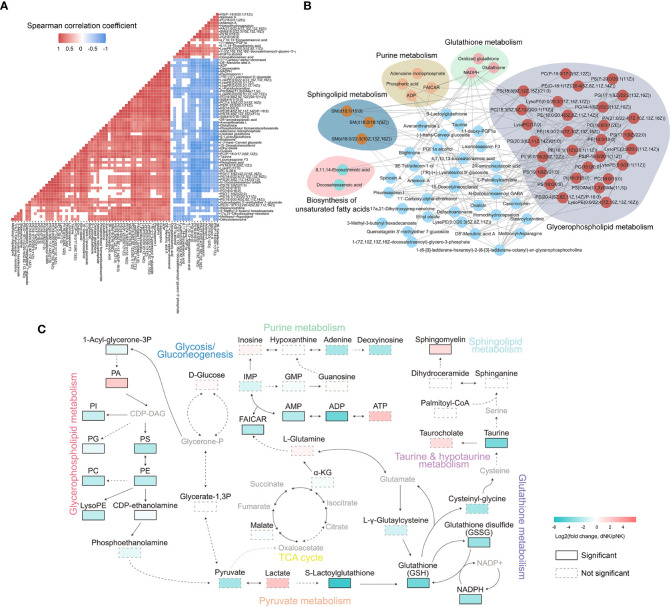
Metabolic network analysis of NK cells. **(A)** Heatmap of Spearman correlation coefficients of differential metabolites between dNK and pNK cells. Only paired metabolites with p-value < 0.05 and absolute correlation coefficient greater than 0.6 were colored. **(B)** Spearman correlation networks of differential metabolites with absolute correlation coefficients greater than 0.95. Solid lines represent positive correlations and dashed lines represent negative correlations. **(C)** Schema of metabolic pathways with select metabolites. Color corresponds to the log2 FC between dNK and pNK (ctrl) cells. Gray nodes represent metabolites that were not detected and borders are color-coded by statistical significance. CDP-DAG, Diacyl glycerol; IMP, inosine monophosphate; GMP, guanosine monophosphate; FAICAR, 1-(5’-Phosphoribosyl)-5-formamido-4-imidazolecarboxamide.

Strong correlations among metabolites suggest that the metabolites have similar roles and belong to related metabolic networks (38). To find potential co-regulatory relationships of differential metabolites, we performed Spearman correlation analysis and constructed a correlation network for all metabolites. In total, 85% of differential metabolites were significantly correlated with each other (**Figure 4A**). Notably, most differential metabolites had strong positive correlations with a threshold of absolute correlation coefficient greater than 0.95 (**Figure 4B**). There was a strong correlation between metabolites in the same metabolic pathway such as glycerophospholipid metabolism (**Figure 4B**). Furthermore, different pathways such as glutathione metabolism and purine metabolism seemed to have a significant positive correlation (**Figures 4A, B**). Overall, our survey demonstrated that the metabolic alterations between dNK and pNK cells are highly coordinated.

To further explore the comprehensive metabolic alterations between dNK and pNK cells, we plotted the schema of metabolic pathways (**Figure 4C**). Previous research has validated that glycolysis and oxidative phosphorylation (OXPHOS) are crucial to immune metabolism in NK cells (39–41). Our data demonstrated that glycolysis and the tricarboxylic acid cycle (TCA) cycle have a slight disturbance, indicating that glycolysis and OXPHOS are not vital in metabolic distinctions between pNK and dNK cells (**Figure 4C**). In glycerophospholipid metabolism, PA significantly accumulated in dNK cells (**Figure 4C**). Most metabolites of glycerophospholipid metabolism significantly decreased and affected pyruvate metabolism, leading to significant downregulation of the downstream glutathione pathway (**Figure 4C**). In addition, decreased NADPH could not donate enough electrons to reduce glutathione (GSH) from glutathione disulfide (GSSG), implying a redox balance shift in dNK cells (**Figure 4C**). Sphingomyelin in sphingolipid metabolism showed a significant increase in dNK cells (**Figure 4C**), consistent with a previous report showing that sphingolipid metabolism is a crucial signal for decidualization (42). Taken together, these results highlight interrelated metabolic network modifications in dNK cells compared to pNK cells.

### Proteomics analysis of dNK and pNK

**Figure 5:**
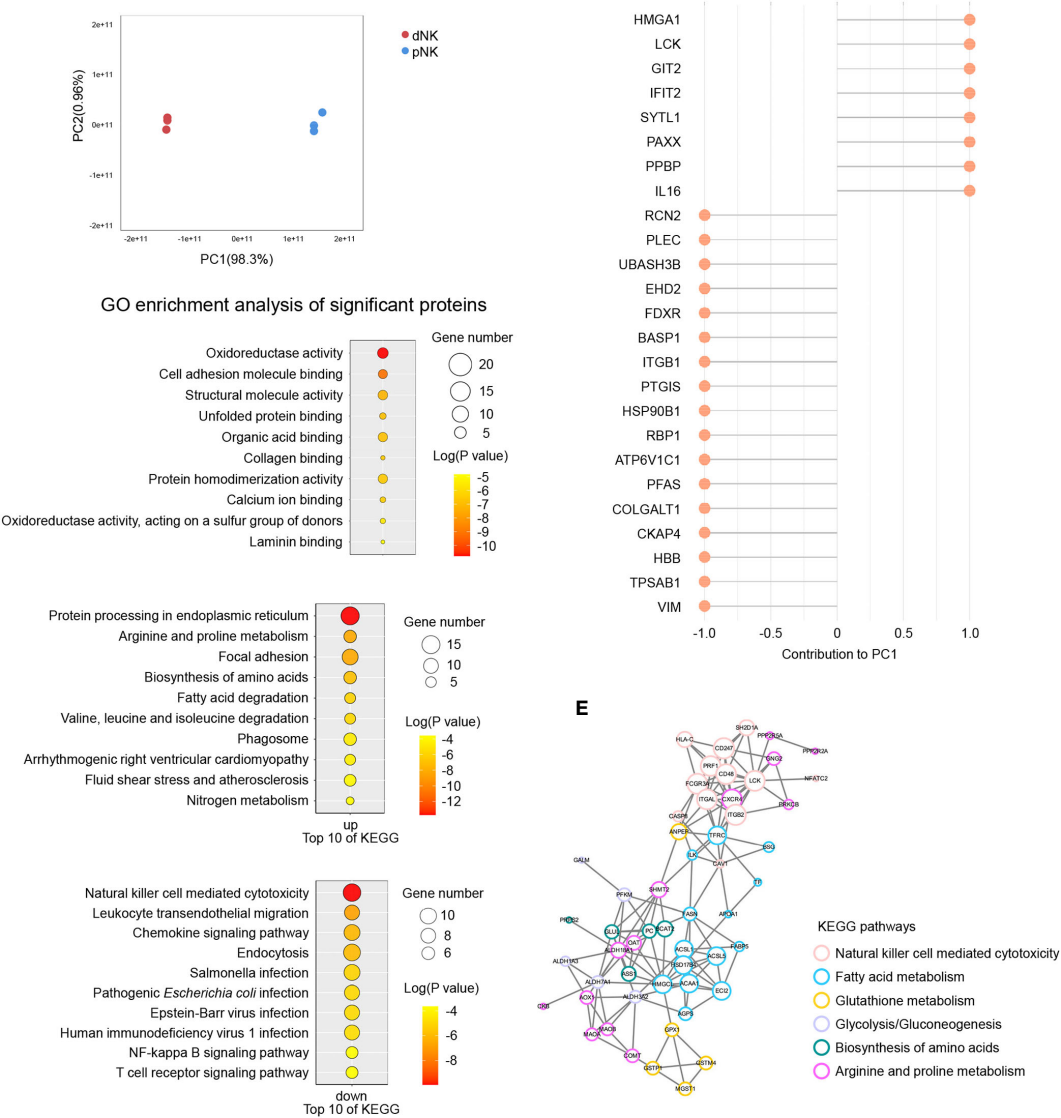
Proteomics analysis of dNK and pNK cells. **(A)** Principal component analysis (PCA) of dNK and pNK (ctrl) cells. Each symbol represents one donor (n=3). **(B)** Top 25 differentially expressed proteins (DEPs) mostly contributing to PC1. **(C)** GO enrichment of DEPs contributing to PC1, |loading| > 0.95. **(D)** KEGG enrichment of all DEPs. **(E)** PPI analysis of DEPs. MCODE was used to calculate the score of each DEP. The size of each circle represents the score.

The above results indicated that metabolism changed significantly between dNK and pNK cells, so we also collected normal dNK and pNK cells and performed proteomics to identify the protein alterations. In total, 2,724 proteins were found and 394 were identified as differentially expressed proteins (DEPs, p-value < 0.05, fold change > 3 or fold change < 1/3) (**Supplementary Figure 2A**). We found 232 upregulated DEPs (fold change > 3) and 162 downregulated DEPs (fold change < 1/3) in dNK cells compared to pNK cells (**Supplementary Figures 2A, B**). Principal component analysis (PCA) showed significant variance in proteomes between dNK and pNK cells, which had a separation in PC1 of 98.3% (**Figure 5A**). We focused on PC1 and displayed the top 25 DEPs that contributed to it (**Figure 5B**). Furthermore, Gene Ontology (GO) enrichment analysis of the DEPs contributing to PC1 (loading > 0.95 or loading < -0.95) showed that DEPs related to oxidoreductase activity were enriched (**Figure 5C**), suggesting that redox equilibria have shifted in dNK cells. In addition, for all GO-enriched DEPs the term oxidoreductase activity was upregulated (**Supplementary Figure 2C**). Some enriched GO terms (supramolecular fiber organization, GTPase regulator activity and actin binding) displayed alterations in the cytoskeleton (**Supplementary Figure 2C**), consistent with previous research showing that dNK cells failed to polarize their microtube to the synapse in contrast to pNK cells (43).

To investigate modified molecular pathways in dNK and pNK cells, we performed KEGG enrichment analysis of all DEPs. NK cell mediated cytotoxicity was the most significantly downregulated pathway in dNK cells (**Figure 5D**). Pathway protein processing in the endoplasmic reticulum had notable upregulation because of active cytokine production in dNK cells (**Figure 5D**). Additionally, leukocyte transendothelial migration appeared to be downregulated because dNK cells are tissue-resident (**Figure 5D**). Apart from the well-known proteins PRF1, CXCR4, HSPA2, etc., most DEPs involved in those three pathways have enormous potential to be investigated in NK cells in the future (**Supplementary Figure 2D**). The most surprising aspect of the KEGG enrichment analysis was DEPs enriched in a set of metabolic pathways, including arginine and proline metabolism, biosynthesis of amino acids, fatty acid metabolism, glutathione metabolism, and glycolysis/gluconeogenesis (**Figure 5D**). Additionally, 110 DEPs were involved in focal adhesion, cytoskeleton remodeling, oxidoreductase activity, and fatty acid metabolism based on pathway enrichment analysis (**Figure 5D**, **Supplementary Figure 2C**). Next, we plotted protein-protein interaction (PPI) networks to find interactions between the NK cell mediated cytotoxicity pathway and metabolic pathways (**Figure 5E**). The PPI network showed that ANPEP and TFRC had strong connections with DEPs in the NK cell mediated cytotoxicity pathway, indicating that metabolism of fatty acids and glutathione influenced NK immune function (**Figure 5E**). Overall, our results indicated various proteome profiles that revealed phenotypic alterations in pNK and dNK cells.

### Integrated metabolomic and proteomic analysis revealed downregulation of glutathione metabolism in dNK compared to pNK cells

**Figure 6:**
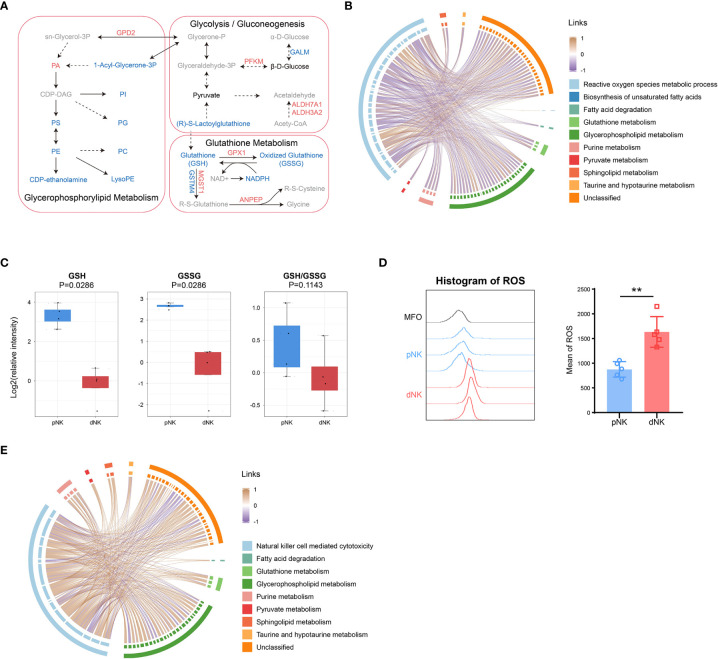
Integration of metabolomics and proteomics of dNK and pNK cells. **(A)** Schema of metabolic pathways (glycolysis/gluconeogenesis, glutathione and glycerophospholipid metabolism) with select metabolites and proteins. Metabolites or proteins significantly upregulated, downregulated, and unchanged were colored in red, blue, and black, respectively. Gray nodes represent metabolites that were not detected. The chord diagrams of all differential metabolites and reactive oxygen species metabolic process **(B)**, and natural killer cell mediated cytotoxicity **(E)**. Spearman correlation coefficients are used as links. **(C)** Relative intensity of metabolites involved in glutathione metabolism in pNK and dNK cells. Statistical analyses were performed by Mann–Whitney U-test. The box plots show the median and 25^th^ and 75^th^ percentiles, with whiskers indicating maximal and minimal values. **(D)** Expression of ROS in dNK and pNK cells. FlowJo was used to plot histogram (left) and GraphPad prism was used for statistical analysis (right). Statistical analyses were performed by two-tailed unpaired Student’s *t*-test and data were presented as mean ± sd.

To understand the comprehensive inner network alterations between dNK and pNK cells, we combined metabolomic and proteomic datasets. The amounts of metabolites altered more than just proteins in glycerophospholipid metabolism, suggesting that upstream metabolites affect more than proteins (**Figure 6A**). Upregulation of GPD2 might result in production of PA, resulting in significant accumulation of PA (**Figure 6A**). Apart from this, glutathione metabolism was altered both in protein and metabolite levels (**Figure 6A**). Increased GSH might enhance cytotoxic functions in NK cells (44–46). We found that lactoylglutathione considerably decreased despite little change in glycolysis or pyruvate metabolism, suggesting that the glutathione source may be limited (**Figures 5C**, **6A**). Glutathione peroxidase 1 (GPX1) was increased in dNK cells, suggesting a compensatory elevation for inner redox environment disruption (**Figure 6A**).

As mentioned above, redox equilibrium might shift in dNK compared to pNK cells. We further explored the correlation between selected DEPs and all differential metabolites. Proteins in the reactive oxygen species (ROS) metabolic process exhibited a negative correlation with common metabolites including glutathione metabolism, implying that proteins in oxidation-reduction (redox) reactions increased activity related to downregulated metabolites (**Figure 6B**). Metabolic reactions are inseparable from redox reactions (47). GSH and GSSG is the major redox pair in cells (48), and the ratio of GSH/GSSG is often used as an indicator of the cellular redox state (49). A notable aspect of our results was that glutathione metabolism was significantly downregulated, which included five downregulated metabolites (**Figure 3A**). Both GSH and GSSG significantly decreased and there was also a downregulated trend in the ratio of GSH/GSSG (**Figure 6C**), indicating alterations in amounts of glutathione metabolism and an imbalance in the conversion of GSH and GSSH. These findings pointed to significant modifications in the redox metabolic pathways between dNK and pNK cells. ROS is the kernel of redox systems and GSH is the major antioxidant for eliminating ROS (44). Therefore, it is reasonable to speculate that dNK has more ROS than pNK cells. To validate this speculation, we detected the ROS contents in dNK and pNK cells using FACS. The data showed that ROS in dNK cells were significantly higher than in pNK cells (**Figure 6D**).

To demonstrate that altered metabolites are interrelated with NK cell functions, we also plotted the correlation chord between proteins in NK cell mediated cytotoxicity and common differential metabolites (**Figure 6E**). The chord chart revealed that cytotoxicity was proportional to metabolite alteration (**Figure 6E**), implying that low cytotoxicity was due to the reduced level of whole cellular metabolites. LCK and PRF1, which were shown to affect cytotoxicity in NK cells, were both significantly downregulated, consistent with the reduced trend of whole metabolite alteration in dNK cells (**Supplementary Figure 2D**). Together, our metabolomics and proteomics data highlight that the inner redox state may affect dNK cell functions.

## Discussion

NK cell immunology has rapidly developed in recent decades and research on it is elucidating NK cell functional fates. Although classical NK phenotypes have indisputably served to identify diverse NK subsets, the phenotypes do not explicitly indicate NK cell functions since phenotypes and functions do not have a one-to-one correspondence (50). In recent years, immunologists have rediscovered the critical role of metabolism in immune cell functions. Recent studies have shown that distinct metabolic features drive NK cell functional potential, which may serve as a reliable way to identify functional fates. Our study provides detailed and exhaustive data regarding the metabolism and protein signatures of dNK cells by using metabolomics and proteomics with independent verification, revealing comprehensive metabolic features. We identified dramatic alteration of glycerophospholipids of dNK cells. Moreover, integrated metabolomics and proteomics data demonstrated the redox disequilibrium and the increased level of ROS in dNK cells. Finally, our data suggested novel perspectives into the metabolic mechanisms of divergent NK cell functions.

Researchers have found that immune cells modify their metabolism to perform various functions (51–54). NK cells modify their metabolic pathways to fulfill certain energy and biosynthetic requirements for various cell functions (55–57). The anti-tumor and antiviral activities of NK cells are compromised by the inhibition of glycolysis and OXPHOS (39, 58, 59). Glutamine withdrawal can suppress IFNγ production and cytotoxicity of NK cells by regulating cMyc (60). Additionally, lipids in the microenvironment appear to change NK metabolism and impair NK cell activities (61, 62). The immunometabolism of pNK cells has been broadly investigated, but the metabolic pathways in tissue-resident NK cells and how cellular metabolism impact their function have not been thoroughly studied. It has been reported that NK cells from peripheral blood differ from liver- and spleen-resident NK cells in the expression profile of nutrient transporters Glut1, CD98 and CD71, consistent with a cell-adaptation to the different nutritional environment in these compartments (63). As for uterus-resident NK cells, Vento-Tormo et al. (26) reported that there are three major subsets of uterine dNK cells (dNK1, dNK2 and dNK3) and predicted that CD39^+^ dNK1 subset can be primed metabolically through increased expression of glycolytic enzymes. Recently, Strunz et al. (64) identified that KIR^+^CD39^+^uterine NK cells presented with increased mitochondrial mass and membrane potential. Here, we performed metabolomics to investigate divergent metabolic pathways in dNK cells distinct from pNK cells. We noticed that the most abundant classes of differential metabolites were lipids and lipid-like molecules. Some studies have shown that lipid metabolism is crucial in coordinating NK cell immunosuppression and impaired under pathological conditions (e.g., obesity and cancer) (65). Our data showed clear reduction of lipids in dNK cells, which seems contrary to earlier findings showing that excessive lipids impaired innate immunity (61). Research on T cells revealed that activated T cells upregulate lipid synthesis and inhibition of fatty acid synthase (FASN) preventing cell death of activated CD4^+^T effector cells (66, 67). Combined with proteomics, a possible explanation for decreased lipids in dNK cells might be that overexpression of FASN (**Supplementary Figure 2D**) inhibits the activation of NK cells. Furthermore, upregulation of the fatty acid degradation pathway may result in decreased lipids in dNK cells.

Glycerophospholipids are nearly the most abundant substances in mammal cellular membranes, and have critical roles in signaling and regulation (68). Glycerophospholipids with long and saturated hydrophobic tails influence membrane fluidity, while polyunsaturated lipids could reduce membrane bending rigidity to promote deformation (37). Here, we reported the alteration of glycerophospholipid metabolism in different NK cell subsets. Total PC, LPC, PE, LPE, PG, LPI, and PS were all significantly downregulated in dNK cells, with only a few metabolites such as PA, PG(16:0/0:0), PC(16:0/3:1(2E)), and PI(16:0/16:0) significantly upregulated. The chain carbon and double-bond analysis emphasized high saturation of glycerophospholipids in dNK cells, suggesting possible difficulty with synapse formation. Moreover, the downregulation of PS might also inhibit dNK cell functions due to the scrambling of PS exposure attenuated NK cell activation (36). Apart from that, PA had an unexpected upregulation in the glycerophospholipids pathway. The reason for this is not clear but it may be due to the unknown block from PA to CDP-DAG. Integrated analysis of proteomics and metabolomics reflected that glycerophospholipid metabolism might have a positive impact on NK cell cytotoxicity. Further work is required to validate the correlation between glycerophospholipids and cytotoxicity in NK cells.

It is commonly accepted that pNK and dNK cells have differential expression of CD56 and CD16 (11, 13). Using high-resolution microarray analyses, Wang et al. found that most dNK cells were immature types with the CD56^bright^CD16^-^T-bet^-^ phenotype, and most of the pNK cells had the CD56^dim^CD16^+^T-bet^+^ mature phenotype (23). Consistent with the literature, our data showed that CD56 was highly expressed and CD16 and T-bet were significantly decreased in dNK cells compared to pNK cells (**Supplementary Figure 1B**). It is worth noting that KEGG enrichment analysis exhibited substantial alterations in some interesting pathways, including focal adhesion, leukocyte transendothelial migration, and chemokine signaling pathway. The downregulation of proteins associated with transendothelial migration and chemokine signal on dNK cells might be because dNK cells, as tissue-resident cells, do not need to cross through the endothelial cells to function like pNK cells. Tight interactions with cells in the decidual microenvironment promoted high expression of focal adhesion proteins on dNK cells.

Our data suggested unexpected alteration of the cytoskeleton and upregulation of protein biosynthesis in dNK cells, which seems to be related to lower cytotoxicity and higher cytokine secretion. Prior studies have noted that immature synapses, which cannot normally release cytotoxic granules, were a physiological mechanism of suppressed cytotoxicity in dNK cells (43). This also accords with our results, which showed prominent enrichment of supramolecular fiber organization and a higher level of cytotoxic granules (granulysin and granzyme A). Furthermore, our findings suggest that the reduction of actin binding did not match the high level of supramolecular fiber organization, providing a reason why synapse maturation was prevented.

Notably, our study described the discrepancy of ROS concentration in the different NK subsets and explored the relationship between redox and cytotoxicity in NK cells. Integrated proteomics and metabolomics demonstrated significant downregulation in glutathione metabolism in dNK cells compared to pNK cells. Although the ratio of GSH/GSSG showed a tendency to decline, the GSH system and the conversion balance between GSH and GSSG exhibited overall downregulation. Downregulation of GSH might impair NK cell functions while high GSH increases cytotoxic functions (45, 46). Since the GSH system is an important antioxidant, we validated the unusually higher ROS concentration in dNK cells, suggesting that redox equilibrium shifted in dNK cells compared to pNK cells. This finding is in line with previous reports that tumor-infiltrating NK cells were impaired by high ROS levels in the tumor microenvironment (69–71). The observed increase in ROS concentration in dNK cells could be attributed to the hypoxic environment in decidua (57, 72–74). Previous studies showed that upregulation of TRX-1 helps GSH maintain a reduced state even though the protection is limited (44). Our proteomics data showed that TXN (TRX-1, data not shown) had nearly a 6-fold increase in dNK cells, indicating a struggle with oxidative stress in dNK cells. Further research should be undertaken to determine the direct relationship between redox and the immunological characteristics of dNK cells.

The interpretation of our findings may be affected by some limitations of our study. First, the range of samples was relatively small and the pregnancy dNK cells and the pNK cell counterparts were not from the same donor. Although it is tempting to reveal comprehensive metabolic alterations between dNK and pNK cells, we cannot rule out the possibility that there is some contribution of immune-metabolic adaptations in pregnancy. Second, untargeted metabolomics cannot provide detailed modification for a specific class of metabolites thus limiting full comprehension of precise metabolic alterations. Third, we did not detect activated NK cells and thus did not compare activated pNK and dNK cells.

Overall, our study identified global metabolic alterations between dNK and pNK cells. Integrated analysis of metabolomics and proteomics revealed the coordinated network of metabolism and phenotype related to NK cell functions. Our data provide a new perspective on dNK immunomodulation, which could be explored in further research. This could be very helpful in creating novel therapies or techniques to ward off specific diseases.

## Data availability statement

The datasets presented in this article are not readily available because the data also forms part of a larger ongoing study. Requests to access the datasets should be directed to FN, fangni@ustc.edu.cn.

## Ethics statement

The studies involving human participants were reviewed and approved by First Affiliated Hospital of the University of Science and Technology of China. The patients/participants provided their written informed consent to participate in this study. Written informed consent was obtained from the individual(s) for the publication of any potentially identifiable images or data included in this article.

## Author contributions

FN, PW, and TL designed the experiments. PW, and TL performed the experiments and data analysis with the help from HZ, MZ, MW, LQ, and YZ. HZ and PW collected blood and decidual samples. FN, PW, and TL wrote the manuscript. FN was responsible for the supervision and project administration. All authors discussed, edited, and approved the final version.
