# Supplementary material for: Unique metabolism and protein expression signature in human decidual NK cells
Source: Front Immunol. 2023 Mar 3;14:1136652. doi: 10.3389/fimmu.2023.1136652 (PMC10020942; doi:10.3389/fimmu.2023.1136652)
Supplement: Supplementary file 1 [file DataSheet_1.docx]

***Supplementary Material***

1. **Supplementary Data**

Supplementary Tables

Supplementary table 1: Differential metabolites between dNK and pNK cells

Supplementary table 2: Differentially expressed proteins between dNK and pNK cells

Supplementary table 3: PCA PC1-loading protein

1. **Supplementary Figures**


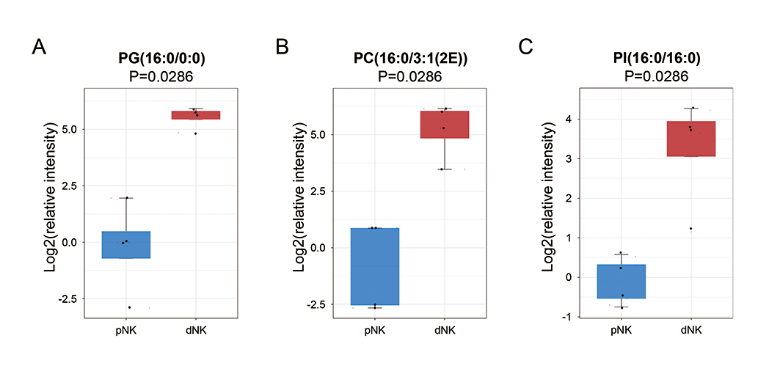


**Supplementary Figure 1 Metabolite relative expression related to Figure 4.** Relative intensity of PG (A), PC (B), and PI (C) in pNK and dNK cells. Statistical analyses were performed by Mann–Whitney U-test. The box plots show the median and 25^th^ and 75^th^ percentiles, with whiskers indicating maximal and minimal values.


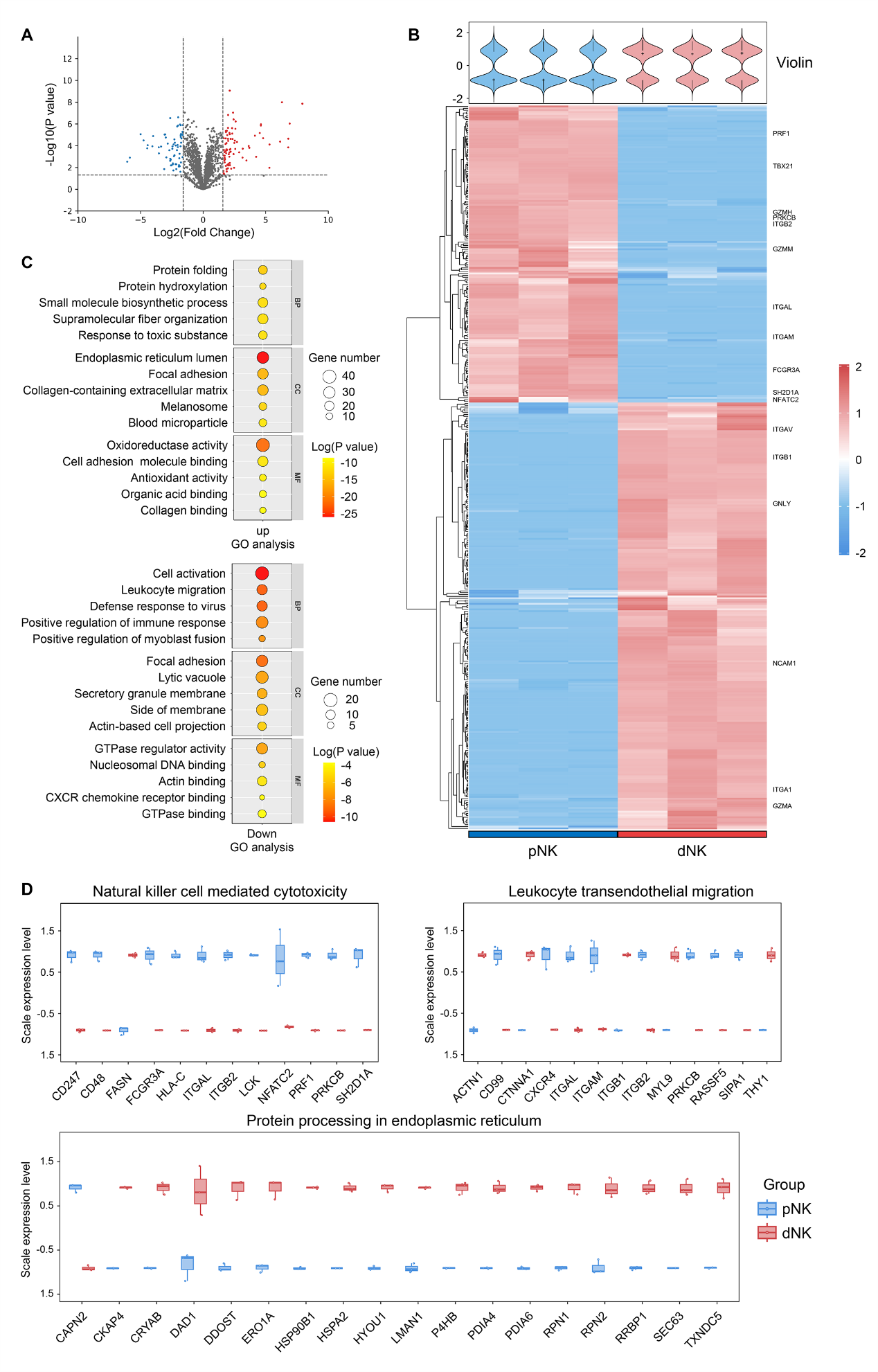


**Supplementary Figure 2 Proteomics analysis of dNK and pNK cells related to Figure 1.** (A) Volcano plot of metabolites detected by untargeted metabolomics in dNK (ctrl) and pNK cells. Significantly upregulated, downregulated (p value < 0.05, fold change (FC) >3 or < 1/3), and unchanged DEPs are colored in red, blue, and gray, respectively. The horizontal line denotes a p-value cutoff of 0.05 and the vertical lines denote an FC of 3 or 1/3. (B) Heatmap of DEPs relative abundance in dNK and pNK (ctrl) cells. (C) GO enrichment of all DEPs. (D) Relative intensity of DEPs involved in selected KEGG pathways in dNK and pNK cells. Statistical analyses were performed by Mann–Whitney U-test. The box plots were visualized as median and 25^th^ and 75^th^ percentiles, with whiskers indicating maximal and minimal values.
